# Causation, mediation and explanation

**DOI:** 10.1093/ije/dyw281

**Published:** 2016-11-18

**Authors:** Neil Pearce, Jan P Vandenbroucke

**Affiliations:** 1 Department of Medical Statistics and Centre for Global NCDs, London School of Hygiene and Tropical Medicine, London, UK and Centre for Public Health Research, Massey University, Wellington, New Zealand; 2 Department of Clinical Epidemiology, Leiden University Medical Center, Leiden, The Netherlands

## *‘Timeo hominem unius libri—*I fear a man of one book’ [attributed to Thomas Aquinas (1225-75)]

This essay review will consider *Explanation in causal inference* by Tyler VanderWeele^1^^,^^2^ in light of a wider discussion about causality, explanation and the future of epidemiology. We will first summarize the book and discuss its strengths and limitations. We will argue that this is a brilliant book about one set of statistical techniques based upon a framework of thinking about mediation and interaction that demonstrates convincingly that these concepts are much more complex than was previously realized in epidemiology and statistics. The preface states that it is aimed directly at people working in applied disciplines of biomedicine and social sciences—with a hope that it will also appeal to statisticians and methodologists. However, it remains within a limited paradigm of causality, and also the applications may be limited. This leads us to question how useful this elegant theoretical approach will be for subject-matter-oriented (public health, clinical) epidemiologists. We conclude that the book is a great reference text, but that careful thought is needed about how to integrate it into the teaching and practice of epidemiology. Most of our remarks are focused on the mediation section, which is the largest and most ground-breaking part.

We have written elsewhere about the limitations (and strengths) of the ‘causal inference’ approach as used by VanderWeele and others, which they have termed the ‘counterfactual approach’ or ‘potential outcomes approach’ (POA). In fact, the latter term is used in several different senses in epidemiology. Often it is introduced as being interchangeable with counterfactual thinking, which does not inherently involve interventions. However, in practice and in terms of statistical theory, the POA is also often used in terms of discussing randomized controlled trials (RCTs) or hypothetical interventions. In this latter use, this approach restricts the questions that epidemiologists may ask, and the study designs that they may use, to questions that are amenable to RCTs (at least in theory). It is this latter approach which we are addressing here, and which we have called the ‘restricted potential outcomes approach’(RPOA).^3^ RPOA theory also restricts the evidence that may be considered acceptable to assess causality, and thereby the evidence that may be considered acceptable for scientific and public health decision making. These restrictions are based on a particular conceptual framework for thinking about causality, and how it should be assessed. We argue that a more reasonable working hypothesis as to the nature of causation and its assessment is that of pragmatic pluralism.^3^ In a sense, the RPOA vision is related to narrow standard evidence hierarchies. We propose broader concepts of causality and causal inference that are pragmatic, eclectic and open to different study design approaches. Ultimately, any approach will be judged as to whether it is useful in terms of identifying the causes of disease in populations; the various theoretical and practical methods that one might use are merely tools and do not define the field.

## What the book does

The title promises to address the topics of causality, inference and explanation. For each of these, what is presented is an elegant, sophisticated, logical, clearly explained but narrow approach. Causality is discussed in terms of potential interventions. Inference (the assessment of causality) is addressed in terms of studies which use the narrow potential outcomes approach (the RPOA). Explanation is then addressed in terms of mediation analysis and statistical interaction assessment in such studies. Within this paradigm, the book is a ‘tour de force’, and the plaudits printed on the back cover are well deserved.

### Classical and modern methods

A ‘traditional’ mediation analysis involved simply adjusting for the intermediate variable (M) and seeing how much the exposure-outcome (A-Y) association changed.^4^ For example, if we were studying the association between smoking and coronary heart disease (CHD) we might try adjusting for blood pressure to see how much of the smoking-CHD association was mediated through blood pressure.^5^ If the smoking-CHD relative risk changed from 2.0 to 1.5, we might say that ‘about half’ of the effect of smoking on CHD was mediated through blood pressure (BP), and this estimate might be quite adequate for assessing the relative importance of this mechanism in public health terms.

**Figure 1.**
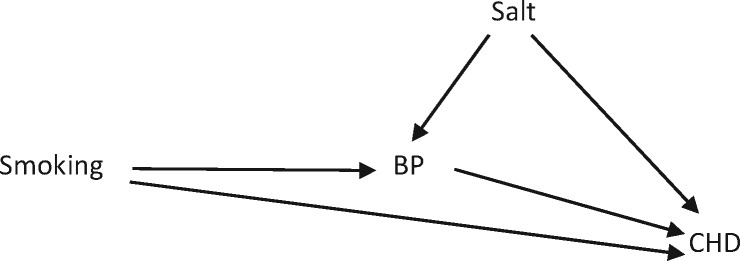
Example of the introduction of collider bias when controlling for blood pressure (BP) to estimate its mediating effect on the association between smoking and coronary heart disease (CHD).

However, these ‘classic’ mediation analyses may be biased if, for example, the blood pressure-CHD (M-Y) association was confounded (e.g. by salt intake); in this situation, controlling for blood pressure would introduce ‘collider bias’. A (hypothetical) example of this is given in Figure 1, building on the previous example: smoking can cause CHD in two ways: (i) it can cause it directly (through some unknown mechanism); and (ii) it can cause it through increasing BP, which in turn increases the risk of CHD. The causal association between smoking and CHD is unconfounded [if you accept that the directed acyclic acyclic graph (DAG) has been drawn correctly], i.e. there is no ‘backdoor pathway’ leading from CHD to smoking (in more traditional terminology, we might say that there is no other factor that is associated with smoking, is predictive of CHD and is not on the causal pathway). Thus, the causal association between smoking and CHD can be estimated without the need to adjust for any confounders. However, if we then attempt to estimate how much of the smoking-CHD association is mediated through blood pressure, problems arise. As described above, we would normally do this by controlling for BP and seeing how much the smoking-CHD relative risk changes. However, when we control for BP, we open up the ‘backdoor pathway’ of CHD-salt-smoking. This happens because BP is a collider on the path from CHD to salt to smoking (there are two arrows going into it). Thus, if we do not control for BP, this backdoor pathway is blocked, but if we do control for BP, then this pathway is opened up because controlling on a collider opens up the path through the collider. One way to think of this is that in this example, smoking and high salt intake are independent, but we are assuming that they are the two main causes of high BP. Adjusting for BP is the same as a stratified analysis. Thus, if we think of the strata in which all the study participants have high BP, among all those who smoke in those strata the probability of having a high salt intake will be lower than in those who do not smoke (because in the non-smokers something else has to cause the high BP and salt is the other causal risk factor in this example). Similarly in the strata of low BP, those who are smokers will be more likely to have a low salt intake and those who are non-smokers to have a higher salt intake. As a consequence, a spurious inverse association is generated between salt and smoking. Thus, the unadjusted smoking-CHD association is unbiased, but the adjusted (for BP) association is biased, and we cannot directly compare the two. In this situation, this ‘standard’ mediation analysis will not produce valid findings. The bias is actually somewhat counterintuitive:^6^ the resulting collider bias is negative, i.e. leads to a underestimation of the direct effect and thus an overestimation of the indirect effect.

Related issues include exposure-mediator interactions and non-linear effects of the exposure on the mediator. Modern mediation analysis addresses these problems (provided that the necessary information is available) by using more sophisticated techniques ^5^ which largely find their origin in Robins’ classic (and at the time, incomprehensible to most of us) 1986 paper.^7^

A similar transformation has occurred in interaction analysis, which has progressed from simple consideration of separate and joint effects^8^ and the distinction between assessing interaction on additive or multiplicative scales,^9^^,^^10^ to the sophisticated methods presented by VanderWeele. However even knowledge from the 1990s, that interaction can be assessed on an additive or multiplicative scale, has not been appreciated in much of the applied literature:^11^ Knol *et al*. reported in 2009 that only 11% of papers that mention interaction give sufficient information to allow the reader to assess both types of interaction (additive or multiplicative).^11^ Nevertheless, the field continues to advance with respect to both mediation and interaction analysis, even though relatively few published studies even use the ‘traditional’ methods appropriately.

VanderWeele has produced ground-breaking publications in the fields of mediation analysis, interaction analysis and the links between them. In Section 1 he outlines the ‘classical’ approach to mediation analysis^12^ and contrasts this (both theoretically and practically) with modern counterfactual approaches.^13^^,^^14^ Section 2 addresses interaction analysis, including the distinctions between statistical interaction, mechanistic interaction and ‘biological’ or ‘functional’ interaction. Section 3 synthesizes the two themes, and clearly shows the various components of causal effects and the different ways of combining them. This includes the decomposition of effects into the controlled direct effect (CDE), the reference interaction (INT_ref_), the mediated interaction (INT_med_) and the pure indirect effect (PIE). These four components can then be combined in various ways to estimate the total indirect effect (TIE), the pure direct effect (PDE), the total direct effect (TDE), the portion eliminated (PE), the portion attributable to interaction (PAI) and so on.

Throughout, the concepts and methods are not only clearly explained but are supported by SAS and Stata code, which will be invaluable to readers wishing to use these techniques. Thus, a key strength of this book is that it has advanced and brought together clearer insights into often obscure problems. Things have come a long way. As a minor historical point, theoretical arguments about interaction and mediation in epidemiology go back to Susser’s 1973 text^4^ which draws on the terminology described by Kendal and Lazarsfeld in 1950^^15^^—long before Baron and Kenny’s 1986 work^12^ that is the starting point of VanderWeele’s introduction to the problem of mediation.

While this book is an enormous intellectual ‘tour de force’, it is also very personal. Issues about the deeper meaning of causation lead VanderWeele in the final chapter to discuss questions of causation as being about questions of meaning. This leads him to give his thoughts about the ultimate cause. In an elegant and restrained argument, amongst others citing Thomas Aquinas (1225-75), he posits that for some people the ultimate cause is named God. This is unusual for a book about epidemiological methods, but scientists do not live in a vacuum and other aspects of a scientist’s life can influence and explain their approach to scientific theory and practice. It speaks for the intellectual honesty and openness of the author that he finds it important to share his ultimate thoughts about causality and the first cause with the reader.

## What doesn’t get (adequately) discussed

So there is much in this book which is innovative and excellent. The problem is what is missing. Whereas the potential biases of the ‘traditional’ approaches to mediation and interaction analyses are clearly stated, there is little acknowledgement or discussion of the theoretical and practical limitations of the ‘modern’ methods which VanderWeele advocates using in their place. This book proposes the use of sophisticated analytical methods in observational studies and implies that by doing so causal inference will be greatly improved. However, we wonder to what extent these analyses really work to solve problems in ‘real world’ studies. There are clear examples (including those given by VanderWeele) where these methods seem to work well, and we also have found them useful.^16^ However, the apparently sophisticated methods are not exempt from the ‘traditional’ problems of epidemiology, namely selection bias, misclassification and residual confounding. Moreover, the ‘sophistication’ of these ‘modern’ methods can obscure these problems. The application of these methods therefore needs (at least) to be accompanied by sensitivity analyses, an issue that rightly receives ample attention in the summary of the book published in this issue of the journal.^2^ The necessity of these analyses is often due to the limitations of the data.

### Misclassification

For mediation, foremost among these is the problem of misclassification which quickly limits the validity of both traditional and modern approaches. Several authors have highlighted this problem.^5^^,^^6^^,^^17^^,^^18^ Misclassification of the mediator may lead to underestimation of the indirect effect, and thus overestimation of the direct effect. VanderWeele considers this but argues that this is only a potential problem and that there exist simple solutions (section 3.5.1). It should be emphasized that misclassification is not just measurement error, but also a matter of identifying the aetiologically relevant exposures, something which is dependent on correct theory and correct and available data.

An example of how intricate and uncertain the analyses might become, is the age-old problem of the constitutional hypothesis by R. A. Fisher, which is mentioned as a relevant example in the summary of the book^2^ and on which a separate paper exists (which we also use here^19^). As VanderWeele *et al.* stated, Fisher proposed that ‘a common genetic cause might explain the association between smoking and lung cancer and thus that smoking might not itself in fact have a causal effect on lung cancer’. As we have noted elsewhere,^3^ Fisher’s hypothesis was effectively refuted by the time trends evidence of rapidly increasing lung cancer incidence and mortality. Nevertheless, VanderWeele *et al*. have investigated the hypothesis and have concluded that Fisher was at least ‘partially right…’.^19^ This conclusion is the result of a mediation analysis in which genes that influence smoking behaviour seemed to have a sizeable direct effect, i.e. by other pathways than ‘smoking intensity’ as measured by ‘number of cigarettes per day’. The latter was apparently the only measure of smoking available; it might lead to considerable misclassification as the relevant exposures might be ‘pack-years’ or other measures of intensity or duration of use, which are not well captured by ‘cigarettes per day’. In fact, the analysis finds that the genetic variants have little influence on the number of cigarettes smoked per day. VanderWeele notes that these variants have been shown not to increase lung cancer in non-smokers, excluding a direct effect in that group. VanderWeele thus uses the example in the book to explain the necessity of interaction analyses, because the gene might only have an effect among smokers (without influencing ‘cigarettes per day’). An alternative interpretation would be that the genetic variants have no effect in non-smokers because they do not increase lung cancer risk (in general), whereas they show an association with lung cancer in smokers because they affect smoking intensity and/or cumulative exposure in ways which are not captured by the mediating variable which was used. They argue that the association ‘seems reasonably robust to measurement error’, but this conclusion applies to the mediating variable that was actually used, which may or may not be valid in this context. Thus, Munafo *et al*. argued that these variants increased the risk of lung cancer largely via tobacco exposure, and that evidence to the contrary ‘is essentially due to imprecision in the measures of tobacco use and exposure’.^20^ Whatever the interpretation of these findings, they illustrate the susceptibility of these analyses both to misclassification and to the choice of mediating variable.

### Confounding

A further potential problem of mediation and interaction analyses, both traditional and modern, is that of residual confounding of the (potential) exposure-outcome effect. It is self-evident that in a study of mediation one should control for not only the confounders of the exposure-outcome relation, but also the confounders of the mediator-outcome relation. However, in practice many analyses are being done on databases where data on both sets of confounders may be missing or misclassified. Of course, in an ideal world with sufficient and correct data, it is possible to draw strong causal conclusions. The problem is that this ideal world is a rare exception. It might only be approached in instances that are a priori relatively confounding-free, such as genetics and adverse drug reactions.

Even in the more straightforward situations, the main ‘strength’ of the modern approach, i.e. the ability to address mediator-outcome confounding, may in many instances be more theoretical than real.^6^ Blakely has shown that such bias (i.e. confounding of the mediator-outcome association) can occur but may involve quite extreme situations, and there has to be very strong confounding of the mediator-outcome association to produce important bias. In theoretical examples this can occur, but in real-life examples^16^^,^^18^ the ‘classical’ approach and the ‘modern’ approach can give very similar findings, although of course this will not always be the case.

### Mendelian randomization

In contrast to the paucity of critical discussion of the potential problems with modern mediation analysis that the book advocates, the Mendelian randomization (MR) approach of using genetic variants as instrumental variables for exposures and mediators is subjected to a detailed critique in section 8.4. It is emphasized that the MR approach depends on strong assumptions: (i) that the genetic marker is associated with the exposure; (ii) that the genetic marker is independent of the outcome, given the exposure and all confounders (measured and unmeasured); and (iii) that the genetic marker is independent of factors (measured and unmeasured) that confound the exposure-outcome association. One could also add the requirements that the genetic marker, exposure and outcome are all measured accurately. It is somewhat unbalanced that the book spends about 20 pages strongly criticizing MR approaches for suffering from precisely the same problems of misclassification and residual confounding that may also wreck mediation analysis. VanderWeele’s concern is that people doing MR studies may fail to assess the extent to which these problems may influence their findings, but (as he in part acknowledges) several key papers on MR have in fact identified these problems and discussed how they can be addressed.^^21–23^^ Furthermore, MR can be used for mediation analysis,^24^^,^^25^ overcoming some of the problems of misclassification of the mediator and/or confounding of the mediator-outcome relationship, which can bias the ‘standard’ mediation analysis approaches proposed by VanderWeele.

## The missing ‘bigger picture’

So even within the limits that it sets itself, we consider that the book falls short in terms of a realistic assessment of the utility of these methods. Our more general concern is that the book misses the ‘bigger picture’ in terms of how it thinks about the core concepts of causality, causal inference and explanation. The book explicitly limits itself to the elegant but limited ‘modern causal inference’ framework. Thus, VanderWeele argues that:

In this book, as well as within the causal inference framework that has come to dominate in statistics, epidemiology, and the social sciences, causation is typically conceived of in terms of contrasts of counterfactual outcomes. These counterfactual outcomes are themselves typically conceived of as the outcomes under hypothetical interventions; and the hypothetical interventions that give rise to counterfactuals usually consist of some human action: for example, a person takes drug A versus drug B’ *…* If we were to abandon the position of trying to relate the meaning of counterfactuals to human freedom, we would be left with the question as to whether counterfactual statements have any meaning at all … It is easier to imagine the rest of the universe being just as it is if a patient took pill A rather than pill B, than it is trying to imagine what else in the universe would have had to be different if the temperature yesterday had been 30 degrees instead of 40.

So if a tree falls in the forest but there was no one there to push it over, then we cannot discuss causality and we cannot identify the causes of the tree falling (even if we have evidence of an earthquake or a storm). More generally, we cannot even begin to address the health effects of important public health issues such as climate change. Nor can we measure the impact of factors that are not interventions (e.g. being diabetic, having a high blood pressure). In fact obesity, hypertension and cholesterol do not appear as ‘causes’ in the book (cholesterol makes one appearance but as an outcome rather than as a cause), and the focus is generally on ‘intervention variables’. Gender does not feature at all although, interestingly, race and ethnicity do feature in several examples (it is questionable whether we can assess mediating factors for something for which we supposedly cannot estimate the causal effect, but that is another debate).

Of course reality and epidemiology are much more interesting and sophisticated than this, and to exclusively adopt this view of causality would be to abandon most of the history and most of the successes of epidemiology. What is presented is a limited vision of epidemiology and what constitutes a cause. VanderWeele proposes amendments to his approach for studying variables that are not amenable to intervention,^26^^,^^27^ but this somewhat difficult ‘fix’ perhaps merely shows the limitations of the paradigm, and has been fundamentally questioned from a philosophical point of view.^3^^,^^28^

## Types of explanation

Similar considerations apply to issues of explanation, which are the major focus of the book. The concept of ‘explanation’ has long played a major role in epidemiology and particularly in the assessment of causality. If we have additional information from other branches of science about why a particular exposure can result in a particular outcome, i.e., its mechanism, this is not only of interest in itself but also can strengthen the evidence that a particular association is causal. Explanatory evidence can take many forms, including qualitative research and a broad range of quantitative research in humans, in other species or *in vitro*. VanderWeele’s approach does not and, more importantly, in some instances cannot take into account other evidence: either because of problems of ‘messy data’ and misclassification, or because the intermediate mechanism cannot be measured numerically. We give two examples of this, one in which the explanatory evidence comes from mechanistic studies in animals rather than humans, and one in which a credible mechanism that is measurable in humans can be assumed.

As a first example, the Monograph Programme of the International Agency for Research on Cancer (IARC) provides one of the leading paradigms for the assessment of causality (i.e. causal inference).^29^ It adopts a broad approach involving evidence from epidemiological studies, on par with the use of animal studies and mechanistic evidence to supplement the epidemiological evidence. For example, TCDD (2,3,7,8-tetrachloro-dibenzo-p-dioxin) was classified in Group 1 (sufficient evidence of carcinogenicity) on the basis of limited evidence in humans and sufficient evidence in animals, together with mechanistic evidence regarding the effects of dioxin on the Ah receptor (AhR). The latter evidence played a major role in explanation of the mechanisms by which dioxin can increase the risk of cancer in general.

A second example involves the major controversy which occurred in the late 1990s about whether third-generation oral contraceptives increased the risk of venous thrombosis more than second-generation contraceptives. Much ink and hot blood flowed when some authors proposed ‘channelling’ of high-risk patients to third-generation contraceptives. Others thought that this was unlikely, as the choice of contraceptives in otherwise healthy young women was generally made without assessment of risk factors—which for venous thrombosis were little known in the 1990s.^30^ The resolution of the debate received a strong impetus from a newly discovered marker of coagulation, APC-resistance. This is augmented in people carrying the factor V Leiden mutation, and is the biochemical mechanism by which factor V Leiden mutation exerts its risk.^31^ Indeed, it was found that APC-resistance is also augmented in women using oral contraceptives, and much more so in women on third-generation contraceptives than on second-contraceptives. Thus, an independently known mechanism of a mutation (factor V Leiden) leading to thrombosis could be linked to a pharmacological risk factor that produced the same elevation of the relevant biochemical characteristic.

The question remains whether these examples of the role of ‘explanation’ would be amenable to statistical mediation analysis of the type that VanderWeele advocates. In the first example, this is not possible because the mechanism (animal and *in vitro* studies) does not lead to a quantifiable intermediate. In the second example, it would probably not be possible to quantify whether all of the effect of third-generation contraceptives occurs via APC-resistance, because of likely substantial misclassification of the mediator and lack of data on mediator-outcome confounders.

These two examples show how the use of explanatory evidence to assess causality often involves a quite different type of reasoning than that assessed in mediation or interaction analyses. It involves considering mechanisms, and different types of scientific evidence. This interlocking of many different parts of evidence led Susan Haack to propose the ‘crossword analogy’ for science: we consider a problem solved when the entries of the crossword fit (wherein each entry has its own support, independent from the others) —but they support each other because they fit in a common explanation.^32^ Although it would in theory be possible to examine these various mechanisms in epidemiological studies and to conduct appropriate mediation analyses, the reality is that the evidence of (causal) associations and of mechanisms of necessity will almost always come from different studies (and perhaps different species). In future we may have statistical methods and rules for combining different types of evidence, but at present causal inference continues to be, and probably always will be, a matter of scientific rather than statistical inference.

## Where does this fit in the future of epidemiology?

As a result of the limits that this book sets itself, it has great depth but little breadth. Perhaps we are asking too much of this text. Perhaps it is not intended to be comprehensive, but only tries to describe a particular set of statistical methods. However, then the book would presumably be titled something less ambitious such as ‘statistical methods for mediation analysis and interaction assessment in observational studies which involve exposures that are potentially manipulable’. Instead, it is staking a claim to a whole field, not just a particular set of methods. Sure, in the first and last chapters of the book (chapters 1 and 16), the author acknowledges that what is being presented is just part of a broader picture. However, this broader picture is ignored in the main body of the book, and it is not made clear how the proposed methods will have their place in this broader picture (chapters 2-15).

The methods presented in this book are very elegant and sophisticated. They are useful in very specific situations (with little misclassification or residual confounding), but it could be argued that those situations are likely to be rare. Furthermore, in an era of epidemiology that seems dominated by interventionist thinking (the RPOA approach), we should keep an open mind to other types of statistical solutions and other developments, such as ‘dynamic influence models’^33^ and complexity theory.^34^ Similarly, standard fixed effects analyses that assess how changes in exposure are associated with changes in outcome^35^ may be better in terms of causal inference than any marginal structural model (MSM) approach when short run change is plausible (Blakely, personal communication). The point is that no set of methods is definitive, and all have strengths and weaknesses; the choice of appropriate methods from the ‘epidemiological toolkit’ will vary according to the exposure, outcome and hypotheses under study.

So where do the methods of VanderWeele ‘fit’ into the bigger picture of epidemiology? Epidemiology starts with the messy world of populations (general, clinical and other), real public health problems and real scientific questions. Like other sciences, it studies a particular ‘object of knowledge’ (in this case, the distribution and determinants of health in populations) and addresses specific scientific and public health questions. Epidemiology and epidemiologists will always use ‘what works’. The various theoretical and practical methods that we may use to address particular scientific and public health problems are merely tools, and do not define the field. The elegant theory and methods of ‘causal inference’ do not define (and restrict the scope of) modern epidemiology any more than the large hadron collider defines (and restricts the scope of) modern physics.

Although the book is very clearly written, the underlying concepts are still difficult, and as a result, these methods are unlikely to be applied routinely by researchers who use epidemiological tools in their studies of public health or clinical problems. This does not mean that these methods should not be used or taught. Sometimes such specialized methods are necessary, and we require the existence of specialists who know how to use them. There are some things that persons with a basic knowledge of epidemiology should not ‘try at home’, just as there are specialized clinical techniques that epidemiologists are unqualified to use. So the existence (and sometimes necessity) of sophisticated statistical techniques is not a problem—it is an opportunity. The problem is when the advocates of sophisticated methods lay claim to an entire field, as the title of this book does.

The danger is that if these methods are presented and taught largely in the abstract, they have the potential to transform epidemiology and severely restrict its practice and vision. It should be acknowledged that both the classic approach and the new theoretical approach have limitations. What is required is a collaborative approach which uses these new developments, in the situations in which they are applicable and appropriate data are available; the corollary is an acknowledgement of when these newer methods are not appropriate and the more traditional methods may be quite adequate (or may even work better in some circumstances). Students and practitioners should be made aware of the existence of these theories and in particular of the likely problems and (im)possibilities in actual data analysis. In practice, very few studies will (even hypothetically) have data available of sufficient accuracy, and including sufficient variables, to enable us to use these methods with any confidence. Their application might often be limited to sensitivity analyses of potential problems and/or ruling out unlikely pathways. In turn, theoreticians should remain keenly aware that their systems may have great value in very particular situations, but that they should not be imposed universally.

In conclusion, a completely transparent and self-contained, logically consistent and rigorous system has been built up, which gives great theoretical insight but is of uncertain practical applicability. What is lacking is an appreciation for the world of epidemiological data, in which issues of misclassification, non-measurement of key exposures and confounding etc. are enormous and have even more strongly detrimental effects on mediation analysis than on ‘main analyses’. What is also lacking is a broader perspective on the field, including an awareness of the broader aetiological theories and debates framing the questions that epidemiologists ask, and the theoretical models they use, which in turn enable mediation and interaction analyses to be conducted. In order for something to be held a mediator or an interactor, and in deciding that some mediation or interaction analysis might be enlightening, there has to be theory about mechanisms and causes that is supported by data different from the data in the mediation analyses itself (see, for a wider treatment of these issues the textbooks of Krieger^36^ and Susser and Stein^37^).

Moreover what is needed is a programme of detailed consideration of applied examples, assessing the strengths and limitations of these and other methods in different situations, bearing in mind the limitations underlying the theory.^38^^,^^39^ Our more general concern remains that if this book redefines what ‘causality’, ‘inference’ and ‘explanation’ are, and what data are needed for a ‘causal’ analysis, then this may have a paralysing effect on epidemiology. The book by VanderWeele will be an important resource for in-depth clarification of theoretical issues and technical statistical analysis, but it should not be the only source of information about problems of causation and explanation. The aphorism ‘Timeo hominem unius libri’ is attributed to Thomas Aquinas, and can be freely translated as ‘I fear the man of one book’. A balanced view of the issues of interaction, mediation and in particular explanation and causality, necessitates that students of epidemiology should read other views alongside this excellent book.

**Conflict of interest:** None declared.

## References

[1] Vanderweele T. Explanation in Causal Inference. New York, NY: Oxford University Press, 2015.

[2] VanderWeele TJ. Explanation in causal inference: developments in mediation and interaction. Int J Epidemiol 2016;45:1904–08. 27864406 10.1093/ije/dyw277 PMC6373498

[3] Vandenbroucke J, Broadbent A, Pearce N. Causality and causal inference in epidemiology—the need for a pluralistic approach. Int J Epidemiol 2016;45:1776–86. 26800751 10.1093/ije/dyv341 PMC5841832

[4] Susser M. Causal Thinking in the Health Sciences. New York, NY: Oxford University Press, 1973.

[5] Richiardi L, Bellocco R, Zugna D. Mediation analysis in epidemiology: methods, interpretation and bias. Int J Epidemiol 2013;42:1511–19. 24019424 10.1093/ije/dyt127

[6] Blakely T. Commentary: Estimating direct and indirect effects—fallible in theory, but in the real world? Int J Epidemiol 2002;31:166–67. 11914315 10.1093/ije/31.1.166

[7] Robins JM. A new approach to causal inference in mortality studies with sustained exposure period - application to control of the healthy worker survivor effect. Math Modelling 1986;7:1393–512.

[8] Pearce N. Analytical implications of epidemiological concepts of interaction. Int J Epidemiol 1989;18:976–80. 2621035 10.1093/ije/18.4.976

[9] Rothman KJ, Greenland S, Walker AM. Concepts of interaction. Am J Epidemiol 1980;112:467–70. 7424895 10.1093/oxfordjournals.aje.a113015

[10] Saracci R. Ineteraction and synergism. Am J Epidemiol 1980;112:465–66. 7424894 10.1093/oxfordjournals.aje.a113014

[11] Knol MJ, Egger M, Scott P, Geerlings ML, Vandenbroucke JP. When one depends on the other reporting of interaction in case-control and cohort studies. Epidemiology 2009;20:161–66. 19034025 10.1097/EDE.0b013e31818f6651

[12] Baron RM, Kenny DA. The moderator mediator variable distinction in social psychological research - conceptual, strategic, and statistical considerations. J Pers Soc Psychol 1986;51:1173–82. 3806354 10.1037//0022-3514.51.6.1173

[13] Seventeenth Conference on Uncertainty in Artificial Intelligence. Direct and Indirect Effects. San Francisco, CA: Morgan Kaufmann, 2001.

[14] Robins JM, Greenland S. Identifiability and exchangeability for direct and indirect effects. Epidemiology 1992;3:143–55. 1576220 10.1097/00001648-199203000-00013

[15] Kendall PL, Lazarsfeld PF. Problems of survey analysis In: Merton RM, Lzarsfeld PF (eds). Continuities in Social Research. Glencoe, IL: Free Press, 1950.

[16] Brewer N, Zugna D, Daniel R, Borman B, Pearce N, Richiardi L. Which factors account for the ethnic inequalities in stage at diagnosis and cervical cancer survival in New Zealand? Cancer Epidemiol 2012;36:E251–57. 22504054 10.1016/j.canep.2012.03.005

[17] Blakely T, McKenzie S, Carter K. Misclassification of the mediator matters when estimating indirect effects. J Epidemiol Community Health 2013;67:458–66. 23386673 10.1136/jech-2012-201813

[18] le Cessie S, Debeij J, Rosendaal FR, Cannegieter SC, Vandenbroucke JP. Quantification of bias in direct effects estimates due to different types of measurement error in the mediator. Epidemiology 2012;23:551–60. 22526092 10.1097/EDE.0b013e318254f5de

[19] VanderWeele TJ, Asomaning K, Tchetgen EJT Genetic variants on 15q25.1, smoking, and lung cancer: an assessment of mediation and interaction. Am J Epidemiol 2012;175:1013–20. 22306564 10.1093/aje/kwr467 PMC3353137

[20] Munafo MR, Timofeeva MN, Morris RW Association between genetic variants on chromosome 15q25 locus and objective measures of tobacco exposure. J Natl Cancer Inst 2012;104(10):740–48. 22534784 10.1093/jnci/djs191 PMC3352832

[21] Lawlor DA, Harbord RM, Sterne JAC, Timpson N, Davey Smith G. Mendelian randomization: Using genes as instruments for making causal inferences in epidemiology. Stat Med 2008;27(8):1133–63. 17886233 10.1002/sim.3034

[22] Davey Smith G, Ebrahim S. ‘Mendelian randomization’: can genetic epidemiology contribute to understanding environmental determinants of disease? Int J Epidemiol 2003;32:1–22. 12689998 10.1093/ije/dyg070

[23] Davey Smith G, Ebrahim S. Mendelian randomization: prospects, potentials, and limitations. Int J Epidemiol 2004;33:30–42. 15075143 10.1093/ije/dyh132

[24] Davey Smith G, Hemani G. Mendelian randomization: genetic anchors for causal inference in epidemiological studies. Hum Mol Genet 2014;23:R89–98. 25064373 10.1093/hmg/ddu328 PMC4170722

[25] Varbo A, Benn M, Davey Smith G, Timpson NJ, Tybjaerg-Hansen A, Nordestgaard BG. Remnant cholesterol, low-density lipoprotein cholesterol, and blood pressure as mediators from obesity to ischemic heart disease. Circ Res 2015;116:665–73. 25411050 10.1161/CIRCRESAHA.116.304846

[26] VanderWeele TJ, Hernan M. Causal effects and natural laws: towards a conceptualization of causal counterfactuals for nonmanipulable exposures, with applications to the effects of race and sex In: Berzuini C, Dawid P, Bernardinell C (eds). Causality: Statistical Perspectives and Applications. Hoboken, NJ: John Wiley, 2012.

[27] VanderWeele TJ, Robinson WR. On the causal interpretation of race in regressions adjusting for confounding and mediating variables. Epidemiology 2014;25:473–84. 24887159 10.1097/EDE.0000000000000105 PMC4125322

[28] Glymour C, Glymour MR. Race and sex are causes. Epidemiology 2014;25:488–90. 24887161 10.1097/EDE.0000000000000122

[29] Pearce N, Blair A, Vineis P IARC monographs: 40 years of evaluating carcinogenic hazards to humans. Environ Health Perspect 2015;123:507–14. 25712798 10.1289/ehp.1409149 PMC4455595

[30] Vandenbroucke JP, Rosing J, Bloemenkamp KWM Oral contraceptives and the risk of venous thrombosis. N Engl J Med 2001;344:1527–35. 11357157 10.1056/NEJM200105173442007

[31] Vandenbroucke JP, Rosendall FR, Bertina RM. Factor V Leiden, oral contraceptives, and deep vein thrombosis In: Khoury MJ, Little J, Burke W (eds). Human Genome Epidemiology. Oxford,UK: Oxford University Press, 2003.

[32] Haack S. Manifesto of a Passionate Moderate. Chicago, IL: Chicago University Press, 1998.

[33] Commenges D, Gegout-Petit A. A general dynamical statistical model with causal interpretation. J R Stat Soc B 2009;71:719–36.

[34] Pearce N, Merletti F. Complexity, simplicity and epidemiology. Int J Epidemiol 2006;35:515–19. 16415326 10.1093/ije/dyi322

[35] Gunasekara FI, Richardson K, Carter K, Blakely T. Fixed effects analysis of repeated measures data. Int J Epidemiol 2014;43:264–69. 24366487 10.1093/ije/dyt221

[36] Krieger N. Epidemiology and the People's Health: Theory and Context. New York, NY: Oxford University Press, 2011.

[37] Susser M, Stein Z. Eras in Epidemiology: The Evolution of Ideas. New York, NY: Oxford University Press, 2009.

[38] Naimi AI. Invited commentary: boundless science - putting natural direct and indirect effects in a clearer empirical context. Am J Epidemiol 2015;182:109–14. 25944884 10.1093/aje/kwv060

[39] Skrondal A. Much ado about interactions. Epidemiology 2014;25:723–26. 25076148 10.1097/EDE.0000000000000093

